# Study of Anthropometry, Range of Motion, and Muscle Strength of Individuals with Spinal Cord Injury or Amputation for the Design of a Driver’s Seat in Large Agricultural Equipment

**DOI:** 10.3390/ijerph192316025

**Published:** 2022-11-30

**Authors:** Yeongjeong Park, Soyoung Yoo, Hyunjoong Kim, Jungkab Choi, Byungchang Son, Juhye Yook

**Affiliations:** 1 Major in Rehabilitation Technology, Graduate School, Korea Nazarene University, Cheonan-si 31172, Republic of Korea; 2 Chungcheongnam-do Assistive Technology Center, Cheonan-si 31172, Republic of Korea; 3 Department of Rehabilitation Technology, Korea Nazarene University, Cheonan-si 31172, Republic of Korea

**Keywords:** spinal cord injury, paraplegia, amputee, anthropometric measurement, agricultural machine, driver’s seat design

## Abstract

This study aims to check and compare the anthropometry, range of motion, and upper limb muscle strength of individuals with spinal cord injury or amputation in order to design a driver’s seat in a large farming machine for them to operate. We analyzed previous studies and derived 24 measurement items essential for designing the driver’s seat. For data collection, we recruited 78 people with spinal cord injury and 46 people with amputation. The collected data were classified into 5th, 25th, 50th, 75th, and 95th percentile groups by disability type and gender, before conducting a comparative analysis. For an in-depth analysis, we performed an independent *t*-test on the samples to compare the individuals with spinal cord injury and the individuals with amputation in terms of disability type and gender. The results showed statistical differences as follows. In the same disability category, male subjects surpassed female subjects. In the same gender category, individuals with amputation surpassed individuals with spinal cord injury. Based on this study’s data and analysis, large farming machines can be designed by reflecting the characteristics of a wide variety of disability types.

## 1. Introduction

According to the 2020 Survey of Disabled Persons in South Korea, 1,289,621 people were reported to have a physical disability. Of them, people with a spinal cord injury account for 8%, and people with amputation account for 11.3%. As for the time of acquiring the physical disability, 97.6% of people were reported to have acquired their disability after birth, which shows that most people with disabilities acquire the status due to injuries they sustained from accidents. To be specific, a leading cause of acquired physical disabilities is musculoskeletal disorders (18.1%), followed by accident-related injuries: automobile accidents (11.5%) and other traumas (27.9%). Moreover, cases of physical disability tend to increase in an aging population [1].

In terms of work injuries in 2021, the rate of fatal injuries among agricultural workers in Korea was 0.80%, an increase of 0.05% from the previous year. This rate of agricultural injury is higher than the workplace injury rate of 0.53% for all industries and the second highest after construction (1.13%), mining (1.3%), logistics (0.92%), and forestry (0.83%) [2]. Furthermore, according to the 2021 Agriculture, Forestry, and Fisheries Survey, the elderly population in rural areas in Korea accounts for 46.8%, which is almost half the rural population [3]. Subsequently, the number of falls and work injuries among elderly farmers while farming or operating farm machines is increasing [4]. As of 2018, agricultural workers who use farming equipment in Korea (3.2%) had a higher rate of work injuries than those who do not use it (2.2%). Among the types of work-related injuries in agriculture, slipping and falling were the most common injuries at 40.8%, and the injuries related to farming equipment were the third most fatal injuries at 12.7% [5].

In a large farming machine, the driver’s seat is the user interface or space where the interaction between human and machine occurs. The driver’s seat essentially comprises a backrest, seat cushion, and armrest. A study on ergonomic design factors for a driver’s seat in a tractor suggested ideal sizes for the seat cushion and backrest, as well as the position of the armrest [6]. The suggestion is for the general population; thus, it is high time that a driver’s seat in farming equipment should be designed with individuals with disabilities in mind.

In designing a seat that requires anthropometry, the National Institute of Technology and Standards has been conducting anthropometric surveys through Size Korea to design products that are easy for Korean people to use in various fields. The Size Korea Project was launched in 1979 and has been implemented eight times every five years. However, this survey project is for the general population. As for the anthropometric survey among individuals with disabilities, only one round of surveys was completed from May 2006 to October 2006 [7]. In order for people with disabilities in Korea to use products that are accessible and safe, it is necessary to take their anthropometric measurements and replace the uncomfortable features of the products designed for the general population [8].

In this study, we took body measurements of individuals with spinal cord injury or amputation to derive the measurement items essential for designing a driver’s seat in large farming equipment and to compare the measurements of individuals with spinal cord injury or amputation.

## 2. Materials and Methods

### 2.1. Deriving the Measurement Items

In this study, we reviewed previous studies on the design of driver’s seats in large farming machines and vehicles. We analyzed a total of 14 studies as follows: “A Study on the Ergonomic Evaluation of Farming Tractor Seat” [9]; “User Analysis for Ergonomic Design of Combine Harvesters” [10]; “Work Analysis for Ergonomic Design of Combine Harvesters” [11]; “A Study on Ergonomic Design Factors for Driver’s Seat of Tractor” [6]; “A Study on the Hand Control for the Self-Driving of Wheelchair Users” [12]; “A Study on the Optimum Driving Posture for Designing Comfortable Driving Work Station” [13]; “Development Trend of Car Seat Technology” [14]; “A Study on the Safety and Human Engineering for the Design Quality-improvement of Vehicle Seats” [15]; “A Study on the Standards of South Korea Type Manual Wheelchair in Accordance with the Human Body Size of Adult” [16]; “Proposition of Korean Type Powered Wheelchair Seat Standards According to Age of the Human Scale” [17]; “Anthropometric and Sports Wheelchair Position Measurement of Athletes with Physical Disabilities for Developing Korean Sports Wheelchair” [18]; “Proposal of Manual Type Wheelchair Standard in Korea” [19]; “Anthropometric Evaluation and Design of Wheelchair” [20]; and “On Anthropometrical Data Acquisition of Human Back Surface for Ergonomic Seat Design” [21]. Based on the measurement items from these studies, we derived a total of 24 measurement items: 18 anthropometric measurement items for designing a driver’s seat in a farming machine, 2 items for measuring the range of motion, and 4 items for measuring upper limb muscle strength. Table 1 shows the details of the measurement items.

The measurement items are related to the design of the driver’s seat as follows. Overhead fist reach, Grip reach(forward) and Foot length are related to the design of the driver’s space. Arm strength push(right elbow 90°), Arm strength push(left elbow 90°), Arm strength pull(right elbow 180°) and Arm strength pull(left elbow 180°) are related to the design of the work station. Chest breadth, Waist breadth, Hip breadth(sitting) and Bideltoid breadth are related to the design of the seat breadth. Sitting height, Cervical height(sitting), Shoulder height(sitting) Elbow height(sitting), Knee height(sitting), Popliteal height(sitting), Foot length, Shoulder-elbow length, Elbow-wrist length, Buttock-popliteal length(sitting) and Buttock-knee length(sitting) are related to the height of the seat, the space under the seat, and the work station design. Thigh clearance(sitting), Range of torso’s flexion and Range of torso’s extension are related to the angle of the backrest. Table 2 shows the relationship between the measurement items, measurement, and design of the farming vehicle seat.

### 2.2. Research Participants and Measurement Instrument

The researchers in charge of measurement had either an academic background or work experience related to disabilities. Prior to conducting the main research, the researchers practiced taking measurements at least five times. The research team comprised two researchers: one taking the measurements and the other assisting with the measuring procedure. The team received two training sessions on how to take measurements. The training was to understand the measurement tools, the anthropometric points, and the measurement methods. The researchers conducted a pilot test in groups.

To recruit research participants with spinal cord injury or amputation, we collaborated with organizations and hospitals that have a registry of people with physical disabilities. Those who saw the recruitment announcement and voluntarily contacted the research team comprised 78 wheelchair users with paraplegia between 26 and 76 years of age (58 men and 20 women) and 46 amputees (34 men and 12 women) between 34 and 69 years of age who are missing one limb (either upper or lower) including disarticulation of a hand or foot. For the individuals with amputation, we took measurements of the normal limb that did not use an assistive device. Table 3 shows the information about the research participants.

As an instrument for taking anthropometric measurements, we used TTM Martin’s human body measuring kit (TSUTSUMI, Tokyo, Japan). To measure range of motion, we used Acumar single and dual digital inclinometers (Lafayette Instrument, Lafayette, Indiana). To measure muscle strength in the upper limbs, we used an ergoFET force gauge (Hoggan Scientific, Salt Lake City, UT, USA) [22]. As the measurement method, we used the method of taking anthropometric measurements, range of motion, and muscle strength to design a farming machine for individuals with amputation as suggested in a previous study [23]. Table 4 shows the details of the measurement instruments.

### 2.3. Analysis Method

The measurement data from the individuals with spinal cord injury (SCI) and the individuals with amputation (Amp.) were classified into 5th, 25th, 50th, 75th, and 95th percentile groups by gender based on the 24 measurement items after eliminating the lowest and highest measurements. Then, for an in-depth analysis on the results of measuring the individuals with spinal cord injury and the individuals with amputation, we performed an independent *t*-test on the samples using IBM SPSS Statistics 25 to compare them in terms of disability type and gender. Prior to conducting the independent *t*-test, we tested the normality. Those who scored above 30 were tested using Kolmogorov–Smirnov; those who scored below 30 were tested using Shapiro–Wilk. The results showed a significance probability of 0.051–0.952 and confirmed normality.

## 3. Results

The measurement data collected from the individuals with spinal cord injury or amputation were classified into 5th, 25th, 50th, 75th, and 95th percentile groups by gender and are presented in Table 5. Then, an independent *t*-test was performed to compare the individuals with spinal cord injury and the individuals with amputation in terms of disability type and gender. The results are presented in Table 6.

Among the measurements of individuals with spinal cord injury, there was a statistically significant difference between men and women in the following measurements: Sitting height (2); Cervical height, sitting (3); Shoulder height, sitting (4); Elbow height, sitting (5); Shoulder-elbow length (10); Elbow-wrist length (11); Buttock-knee length, sitting (13); Foot length (14); Chest breadth (15); Waist breadth (16); Arm strength push, elbow 90° (22); and Arm strength pull, elbow 180° (23–24). In these measurements, the male participants had higher numbers than their female counterparts. The female participants had a significantly higher number in Range of torso’s flexion (19) than the male participants.

Among the measurements of individuals with amputation, there was a statistically significant difference between men and women in the following measurements: Overhead fist reach (1); Sitting height (2); Cervical height, sitting (3); Shoulder height, sitting (4); Knee height, sitting (7); Popliteal height, sitting (8); Grip reach, forward (9); Shoulder-elbow length (10); Elbow-wrist length (11); Buttock-knee length, sitting (13); Foot length (14); Chest breadth (15); Hip breadth, sitting (17); Bideltoid breadth (18); Arm strength push, elbow 90° (22); and Arm strength pull, elbow 180° (23–24). In these measurements, the male participants had a significantly higher number than the female participants.

Among the measurements of individuals with spinal cord injury or amputation, there was a statistically significant difference between men and women in the following measurements: Sitting height (2); Cervical height, sitting (3); Shoulder height, sitting (4): Shoulder-elbow length (10); Elbow-wrist length (11); Buttock-knee length, sitting (13); Foot length (14); Chest breadth (15); Arm strength push, elbow 90° (21–22); and Arm strength pull, elbow 180° (23–24). In these measurements, the male participants had a significantly higher number than the female participants, regardless of disability type.

Among the measurements of the male participants, there was a statistically significant difference based on the disability type in the following measurements: Overhead fist reach (1); Elbow height, sitting (5); Thigh clearance, sitting (6); Hip breadth, sitting (17); Range of torso’s flexion (19); and Range of torso’s extension (20). The individuals with amputation had a higher number than the individuals with spinal cord injury.

Among the measurements of the female participants, there was a statistically significant difference based on disability type in the following measurements: Elbow height (5); Thigh clearance, sitting (6); Grip reach, forward (9); Shoulder-elbow length (10); Range of torso’s flexion (19); and Range of torso’s extension (20). Except for Grip reach, forward (9) and Shoulder-elbow length (10), the individuals with amputation were found to have a higher number than the individuals with spinal cord injury.

Among the measurements of both genders, there was a statistically significant difference based on the disability type in the following measurements: Elbow height (5); Thigh clearance (sitting) (6); Range of torso’s flexion (19); and Range of torso’s extension (20). Except for Range of torso’s flexion (19) and Range of torso’s extension (20), the individuals with amputation were found to have a higher number than the individuals with spinal cord injury regardless of gender.

## 4. Discussion

In this study, we compared the measurement data of individuals with spinal cord injury or amputation with data from the 7th National Anthropometric Survey Report by Size Korea [20], and found that the general population had the highest number in Thigh clearance (sitting), which can be used as a reference for designing the space between the driver’s seat and dashboard. This can be seen as the result of decreased muscle mass in the lower limbs of individuals with spinal cord injury and the result of decreased workouts of the lower limbs among individuals with amputation. In Chest breadth, Waist breadth, and Hip breadth (sitting), which are the measurement items for designing the backrest breadth and the top part of the driver’s seat, the general population had the smallest numbers except for the female participants in the 95th percentile. In Range of torso’s flexion, which is the measurement for designing the angle of the driver’s seat, the general population’s numbers were the highest. This shows that people with disabilities have a small range of motion even though they have a relatively large upper body.

In a previous study on deriving the measurement items for designing a farming vehicle seat [4], the authors derived the following items as factors for the ergonomic design of a tractor seat: Buttock-popliteal length (sitting), Hip breadth (sitting), Chest breadth, Scapula height (sitting), Waist breadth, Hip breadth, and Elbow height (sitting). They also presented anthropometric data of the study subjects from South Korea and the U.S.A. in these measurement items. When we compared the Korean subjects’ anthropometric data from the study [4] with our measurements, we found the following. Measurements of Elbow height (sitting) was the highest in the 5–95th percentile among individuals with amputation regardless of gender. Except for the male participants in the 50th and 95th percentile, individuals with spinal cord injury had the lowest numbers. Measurement of Buttock popliteal length (sitting) was the highest in the 5–95th percentile among the general population regardless of gender, but it was the lowest among individuals with spinal cord injury. Measurement of Chest breadth was the lowest in the 5th percentile among the general population regardless of gender. Measurement of Waist breadth was the lowest among the general population except for the female participants in the 50th and 95th percentile. The male participants in the 95th percentile had the highest numbers among people with spinal cord injury, while the female participants in the 95th percentile had the highest numbers among the general population. Measurement of Hip breadth (sitting) was the lowest in the 5–95th percentile among individuals with spinal cord injury regardless of gender.

As such, the measurements of each measurement item varied based on disability type and gender. Therefore, it is imperative to provide a design of a farming vehicle seat for individuals with spinal cord injury or amputation, different from the one for the general population, or to provide a universal design that can be used regardless of disability status or gender.

## 5. Conclusions

To design a driver’s seat of a large farming vehicle by taking into account specific disability types, we derived anthropometric measurement items and took measurements from individuals with spinal cord injury or amputation. We conducted a comparative analysis on the measurements, and the results are as follows.

- Among the measurements of individuals with spinal cord injury, there was a statistically significant difference between men and women in the following measurements: Sitting height; Cervical height (sitting); Shoulder height (sitting); Elbow height (sitting); Shoulder-elbow length; Elbow-wrist length; Buttock-knee length (sitting); Foot length; Chest breadth; Waist breadth; Arm strength push, elbow 90°; and Arm strength pull, elbow 180°. In these measurements, the male participants had a higher number than the female participants.
- Among the measurements of individuals with amputation, there was a statistically significant difference between men and women in the following measurements: Overhead fist reach; Sitting height; Cervical height (sitting); Shoulder height (sitting); Knee height (sitting); Popliteal height (sitting); Grip reach, forward; Shoulder-elbow length; Elbow-wrist length; Buttock-knee length (sitting); Foot length; Chest breadth; Hip breadth (sitting); Bideltoid breadth; Arm strength push, elbow 90°; and Arm strength pull, elbow 180°. In these measurements, the male participants had a higher number than the female participants.
- Among the measurements of the male participants, there was a statistically significant difference based on the disability type in the following measurements: Overhead fist reach, Elbow height (sitting), Thigh clearance (sitting), Hip breadth (sitting), Range of torso’s flexion, and Range of torso’s extension. In these measurements, individuals with spinal cord injury had a lower number than individuals with amputation.
- Among the measurements of the female participants, there was a statistically significant difference based on the disability type in the following measurements: Elbow height; Thigh clearance (sitting); Grip reach, forward; Shoulder-elbow length; Range of torso’s flexion; and Range of torso’s extension. Except for Grip reach, forward and Shoulder-elbow length, individuals with spinal cord injury were found to have a lower number than individuals with amputation.

In designing the driver’s seat and workstation of a large farm machine, it is essential to consider the operator’s anthropometric data. In determining the angle of the backrest, range of motion data should be considered. Likewise, in designing the dashboard, the operator’s muscle strength should be taken into account. In this study, we only took the anthropometric measurements from individuals with spinal cord injury or amputation. Since the sample size is small, it is difficult to generalize the measurements as the absolute measurements of all people with physical disabilities. In a follow-up study, a researcher can use a larger sample of people with various physical disabilities and take their anthropometric measurements. The measurement items we presented in this study are basic measurements for designing a driver seat. Thus, it is crucial for researchers to conduct a follow-up study to develop a guideline that can be used for designing large farming equipment with physical impairment in mind. Moreover, it is necessary to conduct a study that takes into account various environmental aspects such as mobility and accessibility for individuals with spinal cord injury or amputation during farming activities.

## Figures and Tables

**Table 1:** Deriving the measurement items.

Item/References	[7]	[8]	[9]	[4]	[10]	[11]	[12]	[13]	[14]	[15]	[16]	[17]	[18]	[19]	Selected
Stature	○							○							
Weight								○							
Overhead fist reach, sitting	○				○						○				○
Grip reach; forward, sitting	○				○										○
Sitting height	○				○	○		○			○			○	○
Eye height, sitting	○							○							
Nape height, sitting														○	
Occipital height, sitting														○	
Cervical height, sitting														○	○
Shoulder height, sitting						○					○				○
Scapular height, sitting				○					○	○	○	○	○	○	
Elbow height, sitting	○			○				○	○	○	○	○	○		○
Lumbar height, sitting						○								○	
Sacral height, sitting														○	
Thigh clearance, sitting	○					○					○				○
Knee height, sitting	○					○		○			○				○
Popliteal height, sitting	○							○	○	○	○	○	○		○
High forward reach					○										
Low forward reach					○										
High side reach					○										
Low side reach					○										
Shoulder-elbow length	○					○					○				○
Elbow-wrist length	○										○				○
Elbow-grip length						○									
Buttock-knee length, sitting	○							○			○				○
Buttock-popliteal length, sitting	○			○			○	○	○	○	○	○	○		○
Arm length	○							○							
Hand length	○														
Foot length	○														○
Biacromial breadth								○			○				
Chest breadth	○			○							○			○	○
Waist breadth	○			○										○	○
Elbow to elbow breadth														○	
Hip breadth, sitting	○			○		○	○	○	○	○	○	○	○	○	○
Pelvis breadth											○				
Bideltoid breadth	○													○	○
Foot breadth, horizontal	○														
Chest circumference											○				
Chest depth	○														
Waist depth	○														
Hip depth	○														
Wrist flexion			○												
Wrist extension			○												
Elbow flexion						○									
Neck flexion			○												
Neck extension			○												
Trunk flexion			○			○									○
Trunk extension			○			○									○
Trunk right abduction			○												
Trunk left abduction			○												
Knee flexion						○									
Range of ankle’s dorsiflexion						○									
Range of ankle’s plantarflexion						○									
Shoulder flexion			○												
Shoulder extension			○												
Spinal rotation			○												
Arm strength push		○	○		○										○
Arm strength pull		○	○		○										○
Leg strength push		○	○												

**Table 2:** Relationship between the measurement items, measurement, and design of the farming vehicle seat.

Measurement Items	Number	Measurement	Design of Farming Equipment Seat
Overhead fist reach	1	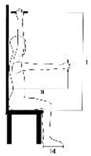	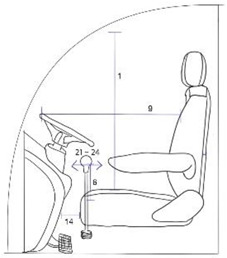
Grip reach, forward	9		
Foot length	14		
Arm strength push, right elbow	21	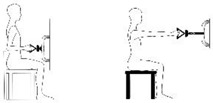	
Arm strength push, left elbow	22		
Arm strength pull, right elbow	23		
Arm strength pull, left elbow	24		
			Driver’s space and work station
Chest breadth	15	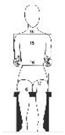	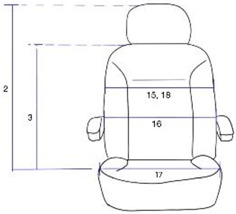
Waist breadth	16		
Hip breadth, sitting	17		
Bideltoid breadth	18		
Sitting height	2	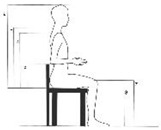	
Cervical height, sitting	3		
Shoulder height, sitting	4		
			Top part of driver’s seat
Elbow height, sitting	5		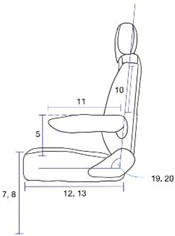
Knee height, sitting	7		
Thigh clearance, sitting	6		
Popliteal height, sitting	8		
Foot length	14		
Shoulder-elbow length	10	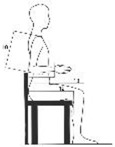	
Elbow-wrist length	11		
Buttock-popliteal length, sitting	12		
Buttock-knee length, sitting	13		
Range of torso flexion, sitting	19		
Range of torso extension, sitting	20		
			Bottom part of driver’s seat and work station

**Table 3:** Research participants.

Disability Type	Gender	Number of Participants	Age (years) Mean ± SD	Height (cm) Mean ± SD	Weight (kg) Mean ± SD
Spinal cord injury	Male	58	51.5 ± 9.2	171.1± 6.7	68.8 ± 12.6
	Female	20	52.9 ± 10.3	162.3 ± 5.2	54.6 ± 8.8
Amputee	Male	34	53.6 ± 10.1	170.6 ± 5.4	73.3 ± 13.0
	Female	12	59.8 ± 6.9	154.6 ± 4.6	60 ± 11.4

**Table 4:** Measurement instruments of anthropometry, range of motion, and upper limb muscle strength.

Measurement Tools	Function	Specification
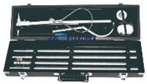 TTM Martin’s human body measuring kit	Anthropometric measurement	Company: TSUTSUMI Co., Ltd. (Japan, Tokyo) Model: TTM Martin’s human body measuring kit Measurement range: 0–1950 mm Margin of error: ±1 mm
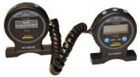 Acumar single and dual digital inclinometers	Range of motion measurement	Company: Lafayette Instrument (Lafayette, Indiana) Model: Acumar single and dual digital inclinometers Measurement range: −180–+180 Margin of error: 1°
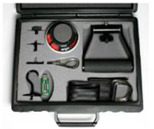 ergoFET force gauge	Pull & Push muscle strength measurement	Company: Hoggan Scientific (Salt Lake City, UT, USA) Model: ergoFET force gauge Measurement range: 0–300 IB Margin of error: ±1%

**Table 5:** Anthropometric measurements and percentile data of individuals with SCI and individuals with amputation.

No	Item (Unit)	Disability Type	Male					Female				
			5% Tile	25% Tile	50% Tile	75% Tile	95% Tile	5% Tile	25% Tile	50% Tile	75% Tile	95% Tile
1	Overhead fist reach (mm)	SCI *	1005.5	1110.1	1156.0	1194.5	1239.7	1027.5	1063.0	1103.3	1141.5	1165.3
		Amp.*	1085.6	1179.5	1207.5	1235.5	1286.5	1061.0	1066.0	1069.0	1107.0	1136.8
2	Sitting height (mm)	SCI	832.8	866.0	889.0	925.8	955.5	807.5	829.8	849.5	861.4	887.5
		Amp.	852.6	888.8	917.5	940.3	972.5	819.7	829.8	839.0	857.3	867.8
3	Cervical height, sitting (mm)	SCI	590.5	617.7	642.0	670.3	688.8	579.7	590.8	618.5	627.5	645.8
		Amp.	610.6	636.5	664.1	679.8	704.8	579.5	593.8	600.6	615.0	622.5
4	Shoulder height, sitting (mm)	SCI	532.8	566.3	585.5	604.0	635.0	521.0	536.3	549.5	570.6	582.0
		Amp.	557.1	577.3	596.5	618.0	648.5	521.8	535.5	551.6	560.3	577.0
5	Elbow height, sitting (mm)	SCI	204.6	227.5	247.0	266.0	283.9	193.8	211.6	219.5	236.8	248.3
		Amp.	237.0	249.8	277.5	298.3	309.0	242.1	250.5	255.5	268.9	281.9
6	Thigh clearance, sitting (mm)	SCI	76.8	89.8	101.0	113.5	138.2	80.0	86.5	99.5	106.3	113.2
		Amp.	106.1	126.5	134.5	145.8	161.0	109.2	113.4	122.0	128.0	140.5
7	Knee height, sitting (mm)	SCI	-	-	-	-	-	-	-	-	-	-
		Amp.	477.0	488.8	502.0	515.3	539.0	432.1	443.3	455.0	471.0	479.2
8	Popliteal height, sitting (mm)	SCI	-	-	-	-	-	-	-	-	-	-
		Amp.	396.0	408.5	426.0	432.3	443.1	356.4	361.5	374.0	381.5	387.3
9	Grip reach, forward (mm)	SCI	653.3	690.8	711.5	747.8	773.0	643.8	684.0	699.0	702.0	718.4
		Amp.	665.0	675.0	697.5	723.5	764.9	624.3	645.0	654.0	667.6	689.2
10	Shoulder-elbow length (mm)	SCI	308.5	327.3	334.9	349.0	361.3	291.9	302.5	319.4	325.5	339.2
		Amp.	309.6	323.5	335.5	348.5	361.4	293.4	298.9	302.0	304.9	312.4
11	Elbow-wrist length (mm)	SCI	232.6	245.0	257.0	265.0	273.3	224.6	234.5	239.0	252.8	256.3
		Amp.	233.1	252.6	255.8	264.3	273.1	215.8	231.7	235.5	242.8	247.2
12	Buttock-popliteal length, sitting (mm)	SCI	419.8	437.8	453.0	485.1	521.3	412.3	420.3	441.0	461.3	474.3
		Amp.	435.7	455.3	478.5	494.3	525.6	427.8	438.3	453.0	459.0	475.3
13	Buttock-knee length, sitting (mm)	SCI	517.8	545.0	566.0	590.0	618.5	494.1	513.5	541.5	568.3	581.2
		Amp.	532.7	552.0	572.0	590.5	619.8	500.3	512.0	539.0	552.0	555.9
14	Foot length (mm)	SCI	225.0	235.0	245.0	250.0	263.4	204.0	213.8	215.0	225.0	232.6
		Amp.	231.2	241.5	247.0	255.3	268.5	208.6	214.8	224.5	228.3	229.6
15	Chest breadth (mm)	SCI	303.7	320.5	333.0	349.0	362.6	269.4	284.0	292.5	311.8	319.0
		Amp.	297.0	305.5	319.0	358.7	370.5	281.8	286.8	287.0	290.7	302.2
16	Waist breadth (mm)	SCI	266.7	297.8	318.0	333.1	360.8	241.8	260.8	280.5	296.4	303.2
		Amp.	282.2	292.5	302.5	322.5	349.6	274.7	280.0	290.0	304.8	333.7
17	Hip breadth, sitting (mm)	SCI	328.9	343.4	373.0	387.0	404.0	329.8	345.2	362.7	381.3	395.2
		Amp.	361.0	439.3	471.5	484.0	516.9	349.7	365.0	370.5	379.5	401.7
18	Bideltoid breadth (mm)	SCI	-	-	-	-	-	-	-	-	-	-
		Amp.	428.8	445.0	468.0	494.3	512.0	395.8	413.1	421.5	440.6	449.6
19	Range of torso’s flexion (°)	SCI	3.0	5.0	10.0	16.0	31.0	14.6	21.5	30.5	34.8	48.6
		Amp.	26.0	37.0	50.0	61.0	76.5	31.8	38.3	52.5	70.0	84.1
20	Range of torso’s extension (°)	SCI	3.0	6.0	9.0	14.8	25.1	3.9	7.3	11.5	18.0	26.6
		Amp.	16.1	20.8	26.0	31.0	37.4	13.3	17.5	23.0	26.0	28.7
21	Arm strength push, right elbow 90° (N)	SCI	71.3	103.2	126.3	155.2	230.5	34.8	55.0	96.9	113.1	119.8
		Amp.	87.2	100.7	124.5	180.3	208.6	35.8	43.1	60.5	82.7	93.8
22	Arm strength push, left elbow 90° (N)	SCI	54.0	100.1	126.6	179.9	227.7	39.4	58.7	80.7	100.5	119.8
		Amp.	71.9	104.1	171.7	203.7	246.0	44.1	52.5	65.1	80.9	87.5
23	Arm strength pull, right elbow 180° (N)	SCI	85.6	136.6	162.9	208.4	280.4	33.5	70.5	84.6	118.6	178.7
		Amp.	116.4	157.5	250.5	316.5	420.6	56.9	65.8	78.7	100.1	118.7
24	Arm strength pull, left elbow 180° (N)	SCI	85.0	127.3	165.4	221.7	291.9	41.9	59.7	82.7	110.7	139.9
		Amp.	110.2	163.2	204.0	314.1	388.1	46.2	77.4	82.3	97.4	118.1

* SCI: individuals with spinal cord injury; Amp.: individuals with amputation.

**Table 6:** Statistical analysis of independent *t*-test by disability type and gender.

Category	No	Item	*t*-Value							
			Disability Type				Gender			
			SCI		Amp.		Male		Female	
			M	F	M	F	SCI	Amp.	SCI	Amp.
Anthropometric measurement	1	Overhead fist reach	2.275		5.093 **		−3.646 **		0.653	
	2	Sitting height	5.128 **		8.020 **		−1.734		0.623	
	3	Cervical height, sitting	3.179 *		5.611 **		−2.611		1.149	
	4	Shoulder height, sitting	3.923 **		4.932 **		−2.263		0.224	
	5	Elbow height, sitting	3.544 **		2.424		−5.066 **		−5.383 **	
	6	Thigh clearance, sitting	1.135		1.950		−7.780 **		−5.168 **	
	7	Knee height, sitting	-		6.878 **		-		-	
	8	Popliteal height, sitting	-		9.093 **		-		-	
	9	Grip reach, forward	2.447		4.285 **		1.032		3.388 *	
	10	Shoulder-elbow length	4.506 **		8.859 **		0.253		3.227 *	
	11	Elbow-wrist length	3.882 **		5.292 **		−0.323		1.679	
	12	Buttock-popliteal length, sitting	2.365		2.502		−2.206		−1.090	
	13	Buttock-knee length, sitting	3.067 *		4.213 **		−0.980		0.706	
	14	Foot length	8.057 **		6.729 **		−2.096		−0.876	
	15	Chest breadth	7.403 **		6.892 **		1.047		0.631	
	16	Waist breadth	5.050 **		1.487		0.990		−1.987	
	17	Hip breadth, sitting	0.782		8.232 **		−9.837 **		−1.316	
	18	Bideltoid breadth	-		4.793 **		-		-	
Range of motion measurement	19	Range of torso’s flexion	−6.597 **		−0.700		−10.813 **		−3.547 *	
	20	Range of torso’s extension	−0.978		1.733		−8.717 **		−2.886 *	
Upper limb muscle strength measurement	21	Arm strength push, right elbow 90°	4.017 **		6.554 **		−0.122		1.905	
	22	Arm strength push, left elbow 90°	5.660 **		7.348 **		−1.292		1.749	
	23	Arm strength pull, right elbow 180°	4.071 **		7.941 **		−3.206		0.780	
	24	Arm strength pull, left elbow 180°	6.226 **		7.516 **		−2.770		0.281	

* *p* ≤ 0.01 ** *p* ≤ 0.001.

## Data Availability

The data used for this study are available upon request.
